# Phase matching as a gate for photon entanglement

**DOI:** 10.1038/srep46115

**Published:** 2017-07-13

**Authors:** A. M. Zheltikov

**Affiliations:** 1 Physics Department, International Laser Center, M.V. Lomonosov Moscow State University, Moscow 119992, Russia; 2 Department of Physics and Astronomy, Texas A&M University, College Station TX 77843, USA; 3 Russian Quantum Center, ul. Novaya 100, Skolkovo, Moscow Region, 143025 Russia; 4 Kazan Quantum Center, A.N. Tupolev Kazan National Research Technical University, Chetaev 18a, 420126 Kazan, Russia

## Abstract

Phase matching is shown to provide a tunable gate that helps discriminate entangled states of light generated by four-wave mixing (FWM) in optical fibers against uncorrelated photons originating from Raman scattering. Two types of such gates are discussed. Phase-matching gates of the first type are possible in the normal dispersion regime, where FWM sidebands can be widely tuned by high-order dispersion management, enhancing the ratio of the entangled-photon output to the Raman noise. The photon-entanglement gates of the second type are created by dual-pump cross-phase-modulation-induced FWM sideband generation and can be tuned by group-velocity mismatch of the pump fields.

Four-wave mixing (FWM) in optical fibers provides a compact, tunable, and efficient source of quantum states of light^1^,^2^,^3^,^4^. Modern fiber technologies lend a vast parameter space to tailor such states^5^,^6^, helping tune their entanglement degree and enabling the generation of factorable photon states^7^. Specifically, photonic crystal fibers (PCFs)^8^, where the dispersion and nonlinearity can be managed by fiber design engineering^9^, have been shown to enable photon-pair generation within a broad range of pump wavelengths^1^,^2^,^3^,^4^,^5^,^10^, offering a unique platform for fiber-based quantum communication and information technologies. Highly birefringent fibers^11^, including specifically designed PCFs^8^, have been found to be instrumental in the generation of polarization-entangled photon pairs, opening the ways toward multipartite entanglement^12^. Frequency conversion via FWM has been demonstrated as a method of ultralow-noise of individual- and entangled-photon-state translation^13^. When combined with appropriate single-mode filtering, FWM in optical fibers can serve as a source of single photons with a high degree of quantum indistinguishability^14^, offering an advantageous framework for quantum information processing, quantum metrology, and quantum key distribution.

Raman scattering has long been recognized as a major physical factor that limits the performance of fiber-optic sources of quantum states of light^15^. Light fields propagating through optical fibers inevitably interact with optical phonons, accumulating noise due to the Raman scattering^16^,^17^. This noise limits soliton squeezing in optical fibers^15^ and degrades the performance of fiber-based sources of nonclassical light, including fiber-optic sources of entangled photon pairs^16^,^17^.

In a broader context of classical nonlinear optics, the interplay between FWM and Raman scattering gives rise to a vast variety of nonlinear-optical field evolution scenarios. In optical fibers, FWM effects have been shown^18^ to dominate over stimulated Raman scattering (SRS) as long as phase matching is satisfied for the FWM process. While for narrowband input fields, provided by pico- and nanosecond input pulses, well-resolved FWM and SRS signatures can often be isolated in broadened output spectra^19^,^20^, femtosecond laser pulses tend to undergo more complicated temporal and spectral transformations, where the FWM dynamics is intertwined with SRS effects, giving rise to octave-spanning supercontinua^21^,^22^, as well as frequency-shifting^23^,^24^ and self-compressing^18^,^25^ soliton transients. In nonlinear Raman spectroscopy^26^ and microscopy^27^, FWM is manifested as a coherent nonresonant background, which generally limits the sensitivity of imaging and spectroscopic measurements, but in certain schemes can also serve as a local oscillator, facilitating a heterodyning of the coherent Raman signal^28^. The nonresonant FWM background in nonlinear Raman spectroscopy and microscopy can be efficiently suppressed by using properly optimized delay times^29^, polarization geometries^30^,^31^, pulse shapes^32^, and phase profiles^33^ of the pump, Stokes, and probe pulses.

Here, we demonstrate that the Raman noise can be radically reduced in fiber-optic FWM-based photon entanglement generation through carefully tailored phase matching, which provides a tunable gate that helps discriminate entangled photon pairs against uncorrelated photons originating from Raman scattering. Two types of phase-matching gates will be considered. Phase-matching gates of the first type are possible, as shown below in this paper, in the normal dispersion regime, where FWM sidebands can be widely tuned by high-order dispersion management, enhancing the ratio of the entangled-photon output to the SpRS noise. The photon-entanglement gates of the second type are created by dual-pump cross-phase-modulation-induced FWM sideband generation and can be tuned by group-velocity mismatch of the pump fields.

## Four-wave mixing as a source of entangled photon pairs

We consider a generic 2*ω*_p_ = *ω*_s_ + *ω*_a_ FWM process where two pump photons of the same frequency, *ω*_p_, give rise to idler and signal photons (also referred to hereinafter as the Stokes and anti-Stokes photons) with frequencies *ω*_s_ and *ω*_a_. In the undepleted-pump approximation, the Hamiltonian *H*_FWM_ that describes all the FWM processes coupling these fields is quadratic^34^ in the Stokes and anti-Stokes field creation and annihilation operators 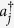 and *a*_*j*_ (*j* = s and a for the Stokes and anti-Stokes fields), defined in such a way as to satisfy the commutation relations 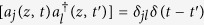. In the Heisenberg picture, the solution to the evolution equations 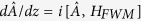 for these operators, 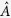 = 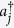, *a*_*j*_, can be written in the input–output form as^35^ 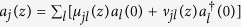, and 
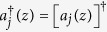
, with *μ(z*) and *ν(z*) being the transfer functions.

In the case when both pump photons are taken from the same pump field, *ω*_p1_ = *ω*_2p_ = *ω*_p_ and 2*ω*_p_ = *ω*_s_ + *ω*_a_, and the FWM Hamiltonian^34^ is 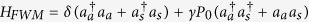, where *P*_0_ is the peak power of the pump, *γ* is the Kerr nonlinearity coefficient, *δ* = Δ*β*/2 + *γP*_0_, Δ*β* = *β*_s_ + *β*_a_ − 2*β*_p_, and *β*_*p*_, *β*_*s*_, and *β*_*a*_ are the propagation constants of the pump, Stokes, and anti-Stokes fields, the solution for 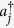 and *a*_*j*_ reduces to^12^


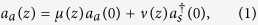



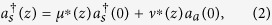


where *μ(z*) = cos(*κz*) + *i(δ*/*κ*)sin(*κz*), *ν(z*) = *i(γP*_0_/*κ*)sin(*κz*),*κ*^2^ = *δ*^2^ − (*γP*_0_)^2^.

With *a*_*j*_ and 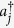 defined by Eqs (1) and (2), the expectation value for the photon number 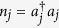 is 〈*n*_*j*_(*z*)〉 = |*ν(z*)|^2^. For a two-mode input vacuum state 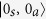, the FWM-sideband output is in the squeezed state^35^


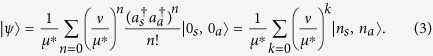


Quantum correlations between the Stokes and anti-Stokes photons are quantified in terms of the cross-correlation coefficient^34^ 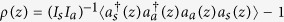. For a pure, Raman-noise-free two-mode squeezed-state FWM output^12^, 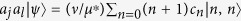 and 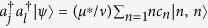, with *c*_*n*_ = (μ^*^)^−1^(*ν*/μ^*^)^*n*^, we find 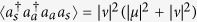 and *I*_*s*_*I*_*a*_ = 〈*n*_*s*_〉〈*n*_*a*_〉 = |*ν*|^4^, leading to the following expression for the cross-correlation coefficient: *ρ*_0_(*z*) = |*μ(z*)|^2^/|*ν(z*)|^2^.

## Raman-effect-induced degradation of photon-pair correlations

The Raman effect is included in the model of FWM sideband generation through the inertial part of the nonlinear-optical response^18^ and through the 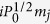 term in the evolution equations for *a*_*j*_ and 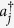 with the Hermitian noise source operator *m*_*j*_^15^ defined in such a way that 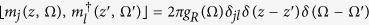, where *g*_R_(Ω) is the Raman gain. With the Raman effect added, the spectral density *S*_*j*_(*z*) of the Stokes (*j* = s) and anti-Stokes (*j* = a) photon flux 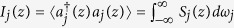 is^17^ 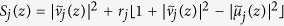, where 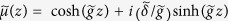, 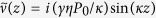, 
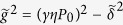
, 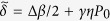, *η* = 1 + *f*_*R*_(*R*_1_ + *R*_2_ − 1), *f*_R_ is the Raman fraction of the nonlinear refractive index, *R*_1_ and *R*_2_ are the Fourier transforms of the isotropic and anisotropic parts of the Raman response, such that *g*_*R*_ = 2*γf*_*R*_Im(*R*_1_ + *R*_2_), *r*_*a*_(*z*) = *n*_*th*_(Ω_*a*_), *r*_*s*_(*z*) = −*n*_*th*_(Ω_*s*_) − 1, Ω_*j*_ = *ω*_*j*_ − *ω*_p_, *n*_*th*_(Ω) = [exp(*ħ*|Ω|/*θ*) − 1]^−1^ is the thermal photon number, *θ* = *k*_B_*T, k*_B_ is the Boltzmann constant, and *T* is the temperature.

Unlike FWM, which can generate strongly correlated Stokes and anti-Stokes photon pairs as a part of the two-mode squeezed-state FWM output of Eq. (3), spontaneous Raman scattering gives rise to uncorrelated Stokes and anti-Stokes photons, which follow a thermal distribution of phonon population *n*_th_(Ω). As a result, the Raman noise decreases correlations between the Stokes and anti-Stokes photons. The degree of this correlation degradation, however, strongly depends on the phase mismatch Δ*β*. This dependence, as shown below in this paper, helps discriminate entangled states of light generated by FWM against uncorrelated photons originating from Raman scattering.

## Phase matching

To understand the significance of phase matching for correlated photon-pair generation, we first consider the case of large phase mismatch, |Δ*β*| ≫ *γP*_0_. In this regime, the spectral density of the photon-pair flux is given by *S*_*j*_(*z*) = *F*_*j*_(*z*) + *S*_*R*_(Ω_*j*_, *z*), with the FWM part of the flux, 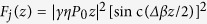, controlled by the signature sinc(Δ*βz*/2) = [sin(Δ*βz*/2)]/(Δ*βz*/2) phase-mismatch factor. When |Δ*βz*| ≫ 1 and *γP*_0_*z* is kept small, *γP*_0_*z* ≪ 1, to avoid an excessive degradation of photon-pair correlations as dictated by *ρ*_0_(*z*) = |*μ(z*)|^2^/|*ν(z*)|^2^, the Raman noise dominates over the FWM photon-pair flux, giving rise to uncorrelated Stokes and anti-Stokes photons with *ρ(z*) ≪ 1.

In the opposite limit, when *δ* is small, FWM sidebands are strongly coupled, giving rise to correlated Stokes and anti-Stokes photons. Moreover, the FWM parametric gain is at its maximum at *δ* = 0, providing the highest efficiency of FWM sideband generation. Within the FWM parametric gain band, i.e., for *δ* < *γP*_0_, the solutions for *a*_*j*_ and 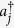 are given by Eqs (1) and (2) with *μ(z*) = cosh(*gz*) + *i(δ*/*g*)sinh(*gz*), 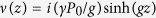, and *g*^2^ = (*γP*_0_)^2^ − *δ*^2^. At the center of the FWM parametric gain band, *δ* = 0, the two-mode squeezed-state output is 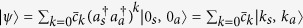, where 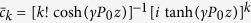.

The cross-correlation coefficient of Raman-noise-contaminated Stokes and anti-Stokes photon pairs in the *γP*_0_*z* ≪ 1 and *δ* = 0 regime is given by^17^


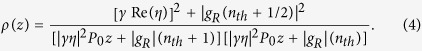


When the Raman noise is negligible, *f*_*R*_ ≪ 1, Eq. (4) reduces to the expression for the Raman-noise-free cross-correlation coefficient *ρ*_0_(*z*) written in the same approximation, i.e., with *δ* = 0 and *γP*_0_*z *≪ 1, leading to *ρ*_0_(*z*) ≈ (*γP*_0_*z*)^−2^. The choice of the nonlinear phase *φ*_nl_ = *γP*_0_*z* is thus a tradeoff between the photon flux *I*_*j*_, which increases with *φ*_nl_ as |*ν(z*)|^2^, and the correlation between the Stokes and anti-Stokes photons, which decreases with *φ*_nl_ even in the absence of the Raman noise as |*μ(z*)|^2^/|*ν(z*)|^2^.

## Discriminating correlated photon pairs against the Raman noise

**Figure 1:**
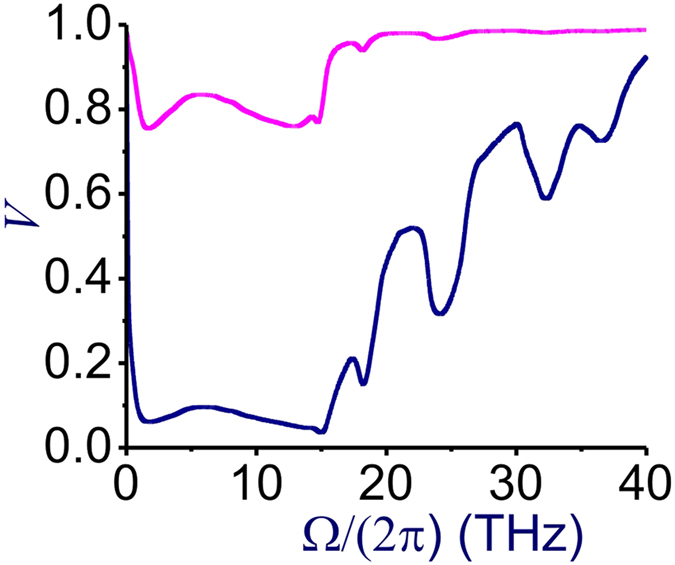
Two-photon interference fringe visibility *V* as a function of the frequency Ω/(2*π*) = (*ω* − *ω*_p_)/(2*π*) for Stokes and anti-Stokes photons generated through the phase-matched 2*ω*_p_ = *ω*_s_ + *ω*_a_ FWM with *δ* = 0, *T* = 300 K, and *γP*_0_*z* = 0.1. (red line) pure FWM with *f*_*R*_ = 0 and (blue line) FWM with the Raman noise, *f*_R_ = 0.18.

We quantify the time–energy entanglement^36^ of the Stokes and anti-Stokes photons in terms of the fringe visibility *V* = *ρ*/(*ρ* + 2) of a two-photon interference pattern, which can be measured, e.g., with the use of an unbalanced Mach–Zehnder interferometer^37^,^38^. Figure 1 shows the parameter *V* plotted as a function of the frequency Ω/(2*π*) = (*ω* − *ω*_p_)/(2*π*) for Stokes and anti-Stokes photons generated through pure FWM with *f*_*R*_ = 0 (red line), as well as through FWM with the Raman noise (blue line). For the highest efficiency of photon-pair generation, FWM is assumed to be ideally phase-matched in both cases, *δ* = 0. The nonlinear phase shift is kept small, *γP*_0_*z* = 0.1, to provide a low-*n*_*j*_ output, which helps avoid an excessive degradation of photon-pair correlations. Parameters of the Raman noise are chosen in such a way as to mimic the Raman effect in silica fibers^17^,^18^: *f*_R_ = 0.18 and the peak Raman gain *g*_R0_ = 6.2 10^–12^ cm/W.

**Figure 2:**
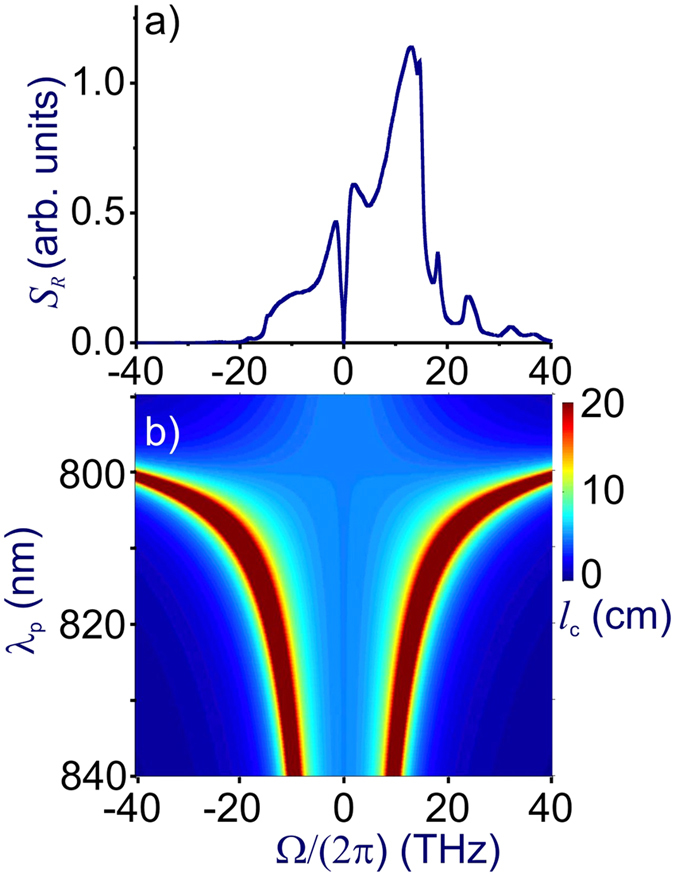
(**a**) The spectral density of the Raman noise *s*_R_(Ω) for fused silica at *T* = 300 K. (**b**) The coherence length *l*_c_ = *π*/|2*δ*| for 2*ω*_p_ = *ω*_s_ + *ω*_a_ FWM with *P*_0_ = 27 W calculated as a function of the pump frequency and the Stokes/anti-Stokes wavelengths *λ*_s,a_ = 2*πc*/*ω*_s,a_.

To understand the influence of Raman scattering on quantum correlations between the Stokes and anti-Stokes photons as a function of the frequency at which phase matching *δ* = 0 is achieved, it is instructive to isolate the spectral density of the Raman noise^15^,^17^ *S*_*R*_(Ω, *z*) = *s*_*R*_(Ω, *z)P*_0_*z*, where *s*_*R*_(Ω) = |*g*_*R*_|[*n*_*th*_(Ω) + *H*(−Ω)] and *H*(Ω) is the Heaviside step function. Figure 1a shows the spectral density of the Raman noise *s*_R_(Ω) for fused silica at *T* = 300 K with the Raman gain profile *g*_R_(Ω) as specified by Stolen *et al*.^39^. As can be seen from this plot, *s*_R_(Ω) is symmetric with respect to Ω = 0, *s*_R_(Ω_s_) ≈ *s*_R_(Ω_a_), only for low Ω (|Ω|/(2*π*) < 1 THz for *T* = 300 K in Fig. 2a), where |Ω|/(2*π*) < *k*_*B*_*T*/*ħ*. In this region, *n*_th_ ∝ 1/|Ω|, giving rise to constant low-frequency noise. For |Ω|/(2*π*) > *k*_*B*_*T*/*ħ*, the Stokes sideband is much more prone to the Raman noise than its anti-Stokes counterpart. The *s*_R_(Ω) profile features a broad peak at Ω/(2*π*) ≈ 13 THz (Fig. 2a), rolling off by more than an order of magnitude for |Ω|/(2*π*) > 20–30 THz.

This behavior of the spectral density of the Raman noise is crucial for understanding the properties of the time–energy entanglement of the Stokes and anti-Stokes photons as quantified by the fringe visibility *V*. In Fig. 1, we plot the *V* parameter as a function of the frequency Ω/(2*π*) for the phase-matched 2*ω*_p_ = *ω*_s_ + *ω*_a_, *δ* = 0, and *γP*_0_*z* = 0.1 FWM process with (blue line) and without (red line) the Raman effect. As one would expect from the spectral profile of *s*_R_(Ω), for low Ω, the Raman noise dramatically reduces the entanglement of the Stokes and anti-Stokes photons. Indeed, for Ω/(2*π*) ranging from approximately 1 to 15 THz, the two-photon interference fringe visibility is very low, *V* < 0.1. In this range, Raman scattering imposes severe limitations on fiber sources of quantum states of light.

As the spectral intensity of the Raman noise decreases beyond Ω/(2*π*) > 20–30 THz, the time–energy entanglement of the Stokes and anti-Stokes photons becomes stronger, approaching, for Ω/(2*π*) > 35–40 THz, the Stokes–anti-Stokes entanglement in pure phase-matched FWM (cf. the blue and red curves in Fig. 1). The entanglement of the Stokes and anti-Stokes outputs of FWM can thus be radically enhanced if the high-Ω FWM photons could be selected with an appropriate spectral filtering.

## Four-wave mixing with a single pump

We are going to show now that such a filter can be provided by finely tuned phase matching in optical fibers. Photonic-crystal fibers, where dispersion can be tailored by fiber structure engineering^8^,^9^, thus enabling a fine adjustment of FWM phase matching, are ideally suited for this purpose^40^. As an example, we consider a PCF with zero group-velocity-dispersion (GVD) wavelength *λ*_z_ ≈ 800 nm and a dispersion profile similar to that provided by a family of commercial, NL-800-series PCFs. Fibers of this type have been shown^21^,^40^ to enable highly efficient parametric FWM pumped by a 760–820 nm Ti: sapphire laser output.

FWM gives rise to parametric sideband generation when the wave number *K* of a harmonic perturbation of a cw solution of the relevant wave equation has a nonzero imaginary part. When *β*_2_ = ∂^2^*β*/∂*ω*^2^ < 0 and higher order dispersion terms involving *β*_*k*_ = ∂^*k*^*β*/∂*ω*^*k*^ with even *k* ≥ 4 are negligible, the dispersion relation for *K* is written as^18^


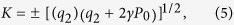


where *q*_*k*_ = *β*_*k*_Ω^*k*^/*k*!. FWM parametric sideband generation is thus possible for any Ω meeting the inequality Ω^2^ < 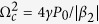. The maximum gain is achieved at the frequency 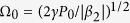, exactly where the phase matching 
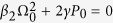
, equivalent to *δ* = 0, is satisfied.

**Figure 3:**
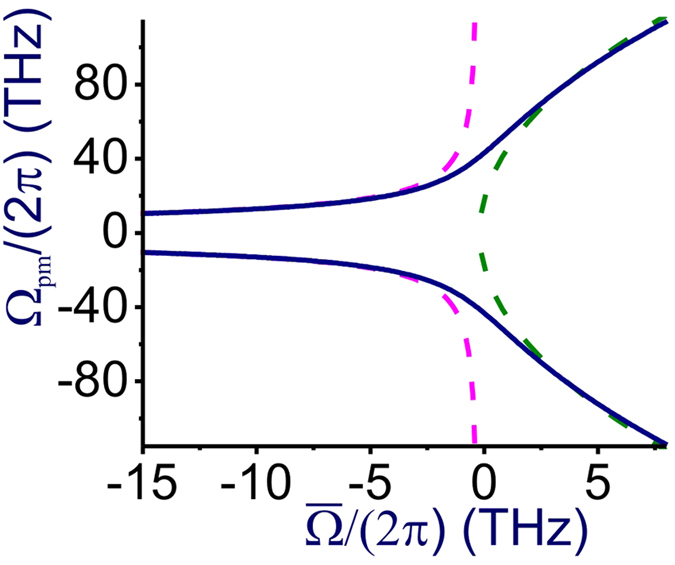
The phase-matching frequency Ω_pm_ as a function of 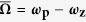 (ω_p_ and ω_z_ are the pump and zero-GVD frequencies) calculated by numerically solving the equation δ = 0 for FWM with *P*_0_ = 27 W in a fiber with the dispersion of an NL-2.4-800 PCF (solid line) versus the approximate solutions Ω_*pm*_ ≈ (2*γP*_0_/|*β*_2_|)^1/2^ in the anomalous-dispersion range (red dashed curve) and Ω_pm_ ≈ (−12*β*_2_/*β*_4_)^1/2^ in the normal-dispersion range (green dashed curve).

Figure 3 compares the phase-matching frequency Ω_pm_ calculated by numerically solving the equation *δ* = 0 for FWM with *P*_0_ = 27 W in a fiber with the dispersion of an NL-2.4-800 PCF (solid line) with the approximate solution Ω_pm_ ≈ 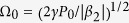 (red dashed curve). As can be seen from this comparison, the approximation Ω_pm_ ≈ Ω_0_ provides a highly accurate prediction for the frequency of phase matching everywhere in the anomalous-GVD range except a narrow region near the zero-GVD frequency *ω*_z_, which corresponds to 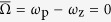 in Fig. 3.

In Fig. 2b, we present a typical map of the coherence length *l*_c_ = *π*/|2*δ*| for 2*ω*_p_ = *ω*_s_ + *ω*_a_ FWM with *P*_0_ = 27 W calculated as a function of the pump frequency and the Stokes/anti-Stokes wavelengths *λ*_s,a_ = 2*πc*/*ω*_s,a_. As an important universal tendency, the FWM phase-matching maps and, hence, the maps of the FWM gain look drastically different for the normal- and anomalous-GVD regions (Figs 2b and 3). When the wavelength of the pump with a peak power *P*_0_ lies in the region of anomalous GVD, where *β*_2_ < 0, a simple 
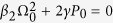
 phase matching is possible for parametric FWM processes, giving rise to two *δ* = 0 phase-matching branches (Figs 2b, 3) with the centers of these parametric gain bands separated from *ω*_p_ by a small spectral interval of 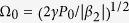.

In the region of normal dispersion, on the other hand, the 
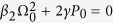
 equation is no longer solvable in the class of real Ω_0_ as *β*_2_ > 0. Still, the *δ* = 0 phase matching is possible due to high-order dispersion, giving rise to two phase-matching branches that lie much further away from *ω*_p_ (Figs 2b, 3). In particular, when high-order dispersion terms *β*_*k*_ with *k* > 4 are negligible, the dispersion equation for *K* is written as^18^,^41^


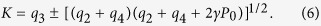


Parametric sideband generation is now possible when 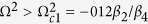 and 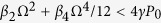. Provided that 
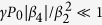
, that is, when the fourth-order dispersion effects can be treated as a small correction within the bandwidth ΔΩ ≈ (2*γP*_0_/*β*_2_)^1/2^, the upper bound of the parametric gain band is given by 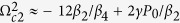. FWM sideband generation is thus confined to a narrow gain band 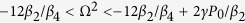, whose bandwidth is on the order of 
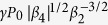
.

As can be seen in Fig. 3, the approximation 
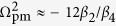
 (green dashed curve) agrees very well with the frequency of phase matching found by numerically solving the *δ* = 0 equation (solid line in Fig. 3) everywhere in the normal-GVD range except a small region near the zero-GVD wavelength. This closed-form approximate expression for Ω_pm_ drastically simplifies the design of fiber sources of entangled photon pairs. Specifically, with 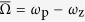 set at just a few terahertz, the entanglement degree of Stokes and anti-Stokes photons, as can be seen from Fig. 1, is increased by more than an order of magnitude. Indeed, with 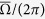 ≈ 0.7 THz, FWM phase matching is achieved at Ω_pm_ ≈ 40 THz (Fig. 3). The two-photon interference fringe visibility for Ω ≈ 40 THz, as can be seen from Fig. 1, is *V* ≈ 0.91, which is more than an order of magnitude higher than the *V* value for Ω ≈ 15 THz. Moreover, with 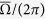 ≈ 7.5 THz, which corresponds to a pump wavelength *λ*_p_ = 2*πc*/*ω*_p_ ≈ 710 nm in the case of a fiber with *λ*_z_ ≈ 800 nm, we find Ω_pm_ ≈ 96 THz (Fig. 3). For a fiber at *T *≈ 25 °C, sideband photons with such a frequency correspond to *ħ*|Ω|/(*k*_*B*_*T*) ≈ 16. The thermal photon number is exponentially small in this regime, *n*_*th*_ ≈ exp(−*ħ*|Ω|/*θ*), providing a strong suppression of the Raman noise in the photon-pair output.

## Four-wave mixing with a dual pump

In dual-pump FWM, cross-phase modulation (XPM) tends to induce energy transfer from one of the pump fields to the sidebands of the other pump^18^,^42^, giving rise to an exponential buildup of sidebands *ω*_1,2_ ± Ω around the central frequency *ω*_*k*_ (*k* = 1, 2) of each of the pump fields. The domains of this XPM-induced parametric gain and their central frequencies Ω_0_ are defined by the dispersion equation^42^,^43^


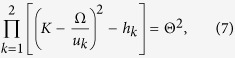


where 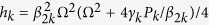, 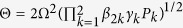, 
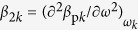
, *P*_*k*_*, ω*_*k*_, *u*_*k*_, and *β*_p*k*_ are the peak power, the central frequency, the group velocity, and the propagation constants of the first (*k* = 1) and second (*k* = 2) pump fields, and *γ*_*k*_ is the nonlinear coefficient at the frequency *ω*_*k*_.

The buildup of XPM-induced sidebands *ω*_1,2_ ± Ω is controlled by the gain *g* = 2 Im*K*, which can be found by solving the quartic equation (7). With Θ = 0, the solution to this equation reduces to 
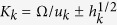
, where *k* = 1, 2. Each of these solutions is equivalent to the solution of Eq. (5), corresponding to a decoupled parametric sideband generation by each of the pump fields.

**Figure 4:**
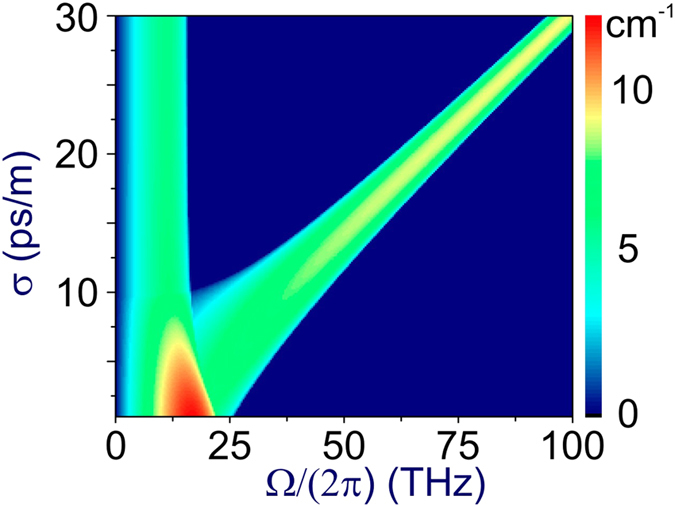
The XPM-induced parametric gain *g* calculated by numerically solving Eq. (7) as a function of Ω and *σ* for fiber pumped by a two-color field consisting of the 1.25 μm Cr: forsterite laser output and its second harmonic with *β*_21_ ≈ − 0.115 ps^2^/m, *β*_22_ ≈ 0.016 ps^2^/m, and *γ*_1_*P*_1_ + *γ*_2_*P*_2_ ≈ 5 cm^−1^.

In a more general scenario, Θ ≠ 0, the two pump fields and their sidebands are coupled by XPM. Both the gain bands and the gain controlling the buildup of XPM-induced sidebands can be tuned in this scheme by varying the frequencies and the peak powers of both pump fields, as well as by tailoring fiber dispersion and nonlinearity. As a typical example, Fig. 4 shows the XPM-induced parametric gain *g* calculated by numerically solving Eq. (7) as a function of Ω and *σ* for the conditions of experiments^44^,^45^, which have demonstrated efficient XPM-induced sideband generation in a PCF pumped by a two-color field consisting of the 1.25 μm Cr: forsterite laser output and its second harmonic. The fundamental-frequency output of a Cr: forsterite laser in this scheme falls in the range of anomalous GVD, with *β*_21_ ≈ −0.115 ps^2^/m, while the second-harmonic pump lies in the region of normal GVD, with *β*_22_ ≈ 0.016 ps^2^/m. The nonlinear coupling constant is *γ*_1_*P*_1_ + *γ*_2_*P*_2_ ≈ 5 cm^−1^.

The g(Ω,*σ*) map in Fig. 4 exhibits two clearly resolved parametric gain bands. The high-frequency band is seen to shift almost linearly with the group-velocity mismatch (GVM) of the pump fields 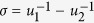, while the low-frequency band is largely independent of the GVM. The former gain band is of special interest for the generation of entangled photon pairs, as it delivers photons with large frequency offsets Ω, thus helping reduce the flux of uncorrelated photons due to the Raman effect.

Both the low- and high-frequency parametric bands seen in Fig. 4 have been studied earlier^20^,^28^ by means of numerical analysis of Eq. (7). As an important empirical result, such an analysis confirms that, for sufficiently large *σ*, the frequency shift of the high-Ω gain band grows linearly with the GVM *σ* of the pump pulses. We show below in this section that some of the key properties of XPM-induced FWM gain bands can be qualitatively understood in terms of phase matching, thus suggesting physically transparent design rules for fiber sources of entangled photon pairs.

With this goal in mind, we set *γ*_1_ ≈ *γ*_2_ = *γ* and approximate the propagation constants of the *ω*_1_ + Ω and *ω*_2_ − Ω sidebands as *β*_*k*±_ ≈ *β*_*k*_ ± Ω/*u*_*k*_ + *β*_2*k*_Ω^2^/2 + 2*γ(P*_1_ + *P*_2_). The phase-matching condition for the *ω*_1_ + *ω*_2_ = (*ω*_1_ + Ω) + (*ω*_2_ − Ω) XPM-coupled FWM sideband generation is then written as


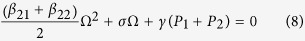


The solution to this equation is


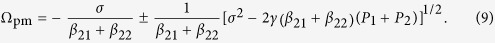


In the case of low pump peak powers, 2*γ(P*_1_ + *P*_2_) ≪ *σ*^2^/|*β*_21_ + *β*_22_|, Eq. (9) gives


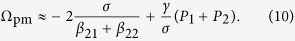


With *γ(P*_1_ + *P*_2_) = 0 and *β*_21_ ≈ *β*_22_ = *β*_2_, Eq. (10) fully recovers the empirical result of the earlier numerical studies^20^,^28^, Ω_pm_ ≈ *σ*/|*β*_2_|. In a more general case of nonzero, but low nonlinearity, *σ*^2^/|*β*_21_ + *β*_22_| ≫ *γ(P*_1_ + *P*_2_) ≠ 0 and *β*_21_ ≠ *β*_22_, the frequency shift of the considered parametric gain band, as can be seen from Eq. (10), is still a linear function of *σ*. In particular, for the parameters of the fiber and the pump in Fig. 4, |*β*_21_ + *β*_22_| ≈ 0.1 ps^2^/m and 2*γ*|*β*_21_ + *β*_22_|(*P*_1_ + *P*_2_) ≈ 100 ps^2^/m^2^, the approximation of Eq. (10) is valid for GVMs *σ* > 10 ps/m. Specifically, for *σ* ≈ 30 ps/m, Eq. (10) predicts Ω_pm_/(2*π*) ≈ 100 THz, which agrees very well with numerical calculations in Fig. 4. For sideband photons with Ω/(2*π*) ≈ 100 THz in a fiber at *T* ≈ 25 °C, *ħ*|Ω|/(*k*_*B*_*T*) ≈ 16, and the thermal photon number is exponentially small, *n*_*th*_ ≈ exp(−*ħ*|Ω|/*θ*), leading to a strong suppression of the Raman noise in the photon-pair output.

In the context of correlated photon-pair generation, Eq. (9) provides a closed-form approximate expression that radically simplifies the design of fiber sources of entangled photon pairs. Specifically, with |*β*_21_ + *β*_22_|/2 ≈ 0.01 ps^2^/m, *σ* ≈ 2.5 ps/m, and *γ(P*_1_ + *P*_2_) ≪ 2*σ*^2^/|*β*_21_ + *β*_22_|, the maximum gain of XPM-induced sideband generation in dual-pump FWm is achieved at Ω_pm_ ≈ 40 THz. At this frequency, the time–energy photon-pair entanglement parameter, *V* ≈ 0.91, is more than an order of magnitude higher (Fig. 1) than the *V* parameter for Ω ≈ 15 THz.

Notably, with *γ(P*_1_ + *P*_2_) ≪ *σ*^2^/|*β*_21_ + *β*_22_|, the frequency shift of the high-frequency gain band in XPM-induced FWM sideband generation, as can be seen from Eqs (9) and (10), is almost independent of the pump peak power. The flux of FWM photons can thus be adjusted to avoid photon-pair correlation degradation (see Section 5), independently of the frequency of FWM photon pairs Ω, which helps discriminate correlated FWM photon pairs against uncorrelated Raman photon pairs. As dual-pump FWM offers a vast variety of polarization and spatial-mode arrangements for multiple sideband generation in optical fibers^12^, GVM-controlled phase-matching filter in such schemes is ideally suited for low-noise multipartite photon entanglement creation.

In its general, polarization-nondegenerate version, the dual-pump FWM scheme considered in this section gives rise to multiple sideband pairs, which can be coupled to each other by the Kerr-type optical nonlinearity^18^. The effect that the resulting correlations have on the quantum properties of sideband pairs is, however, drastically different from the effects induced by the Raman scattering. While the Raman-induced sidebands are not correlated as they build up from the noise that follows a thermal distribution of phonon population *n*_th_(Ω), the manifold of FWM processes in orthogonal polarization modes of the fiber give rise to strongly correlated Stokes and anti-Stokes photon pairs, enabling the generation of multipartite entanglement. Indeed, when the peak power of both pump fields in dual-pump FWM is *P*_0_ and the input is a four-mode vacuum state, 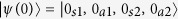, involving two modes *qj (j* = 1, 2) of Stokes and anti-Stokes (*q* = s, a) vacuum fields, the FWM-sideband four-mode output in the *gz* ≪ 1 regime, as shown in the earlier work^12^, is in the squeezed state 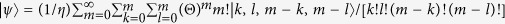, where *η* = 1 − *iγP*_0_*z*, Θ = *iγP*_0_*z*/(2*η*), and 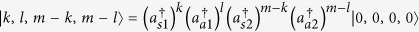. Such states, as elegantly demonstrated by McKinstrie *et al*.^12^, display distinctly identifiable signatures of multipartite entanglement.

## Conclusion

We have shown that phase matching can provide a tunable gate that helps discriminate entangled states of light generated by four-wave mixing in optical fibers against uncorrelated photons originating from Raman scattering. Two types of such gates are discussed. Phase-matching gates of the first type are possible in the normal dispersion regime, where FWM sidebands can be widely tuned by high-order dispersion management, enhancing the ratio of the entangled-photon output to the Raman noise. The photon-entanglement gates of the second type are created by dual-pump cross-phase-modulation-induced FWM sideband generation and can be tuned by group-velocity mismatch of the pump fields.

## Additional Information

**How to cite this article**: Zheltikov, A. M. Phase matching as a gate for photon entanglement. *Sci. Rep.* **7**, 46115; doi: 10.1038/srep46115 (2017).

**Publisher's note:** Springer Nature remains neutral with regard to jurisdictional claims in published maps and institutional affiliations.
